# Why p-OMe- and p-Cl-β-Methylphenethylamines Display Distinct Activities upon MAO-B Binding

**DOI:** 10.1371/journal.pone.0154989

**Published:** 2016-05-06

**Authors:** Angélica Fierro, Dale E. Edmondson, Cristian Celis-Barros, Marco Rebolledo-Fuentes, Gerald Zapata-Torres

**Affiliations:** 1 Facultad de Química, Departamento de Química Orgánica, Pontificia Universidad Católica de Chile, Santiago, Chile; 2 Departments of Chemistry and Biochemistry, Emory University, Atlanta, GA, United States of America; 3 Universidad Andres Bello, Facultad de Ciencias Exactas, Departamento de Ciencias Quimicas, Santiago, Chile; 4 Department of Chemistry, Faculty of Sciences, University of Chile, Santiago, Chile; 5 Faculty of Chemical and Pharmaceutical Sciences, University of Chile, Santiago, Chile; Wageningen University, NETHERLANDS

## Abstract

Despite their structural and chemical commonalities, p-chloro-β-methylphenethylamine and p-methoxy-β-methylphenethylamine display distinct inhibitory and substrate activities upon MAO-B binding. Density Functional Theory (DFT) quantum chemical calculations reveal that β-methylation and para-substitution underpin the observed activities sustained by calculated transition state energy barriers, attained conformations and key differences in their interactions in the enzyme’s substrate binding site. Although both compounds meet substrate requirements, it is clear that β-methylation along with the physicochemical features of the para-substituents on the aromatic ring determine the activity of these compounds upon binding to the MAO B-isoform. While data for a larger set of compounds might lend generality to our conclusions, our experimental and theoretical results strongly suggest that the contrasting activities displayed depend on the conformations adopted by these compounds when they bind to the enzyme.

## Introduction

The biogenic amine levels in human cells are controlled in part by their oxidation by ubiquitous enzymes known as monoamine oxidases (MAOs). Since the discovery of the antidepressant activities of certain molecules acting as MAO inhibitors (MAOi) around 1950 [1], these enzymes have been associated with neurological and neurodegenerative pathologies such as depression, Parkinson’s and Alzheimer’s diseases [2]. These enzymes, inserted in the outer membrane of mitochondria, use flavin adenine dinucleotide (FAD) as a cofactor [3]. Even though the clinically most relevant endogenous substrates of MAOs are serotonin (5-HT) and dopamine (DA), these enzymes are able to oxidize other monoamines such as benzylamine (BA), phenylethylamine (PEA) and their derivatives [4–8]. Human MAO exists in two isoforms, known as A and B, sharing ca. 70% of sequence identity. Although several MAO crystal structures are available [9–22], the mechanisms of selectivity regarding substrates and inhibitors for each isoform have not yet reached a consensus. New insights regarding the possible catalytic mechanisms for the oxidative deamination of neurotransmitter amines by monoamine oxidase B have recently become available [23–26]. Earlier investigations focused largely on the active site of MAOs, located in a cavity lined mainly by hydrophobic residues [27, 28]. A special sector of this active site consists of the so-called “aromatic cage” which contains the FAD cofactor and two tyrosine residues with their rings perpendicular to the isoalloxazine moiety of FAD. It is known that many MAO substrates become inhibitors when they are α-methylated, and most of these products inhibit MAO-A selectively. In contrast, β-methylation of MAO substrates often generates selective MAO-B inhibitors [29]. Almost 30 years ago, Kinemuchi et al. [30] studied 5-fluoro-α-methyltryptamine (5-FMT) and p-chloro-β-methylphenethylamine (p-CMP). These authors found that both molecules act as selective and reversible inhibitors of MAO-A and MAO-B, respectively. Later results by Kim et al. [31] confirmed that p-CMP was a short-acting, probably reversible, MAO-B selective inhibitor. In this article, we report the interesting change in the biological activity displayed by a couple of β-methylphenylalkylamines, where the inhibitor p-CMP becomes a substrate when a methoxyl group replaces its para-chloro substituent.

## Materials and Methods

### Biological evaluation

MAO-B was expressed in *Pichia pastoris* and purified as described previously by Newton-Vinson *et al*. [32] The final step in the preparation of MAO-B used 50 mM potassium phosphate buffer pH 7.4, 50% glycerol, and 0.8% n-octyl-β-d-glucopyranoside (w/v). MAO activity assays were performed by monitoring the rate of product formation over time at 25°C using a Perkin-Elmer Lambda 2 spectrophotometer. p-Methoxy-β-methylphenethylamine oxidation was monitored spectrometrically using the horseradish peroxidase-coupled Amplex Red assay (Δɛ = 54 000 M^−1^•cm^−1^, λ = 560 nm). A similar experiment was carried out with kynuramine (Δɛ = 12 000 M^-1^•cm^−1^, λ = 316 nm) to obtain a control value by monitoring its oxidation.

### General methods

^1^H and ^13^C NMR spectra were recorded on a Bruker Avance 500 MHz spectrometer at 300 K. Coupling constants in Hz were measured from-one dimensional spectra. HRMS-ESI analyses were carried out using a Thermo Scientific Exactive Plus Orbitrap spectrometer with a constant nebulizer temperature of 250°C. The experiment was carried out in positive ion mode at high resolution (resolving power: 140,000 (full width half-maximum peak width at m/z 300, R_fwhm_). The samples were infused directly into the ESI source using a syringe pump at flow rates of 5 μL min^-1^. All chemicals were reagent grade and used without further purification (S1 Fig).

#### 2-(4-Methoxyphenyl)propan-1-aminium chloride

1 g (0,006 mol) of 4-methoxynitrostyrene was dissolved in 50 mL of dry tetrahydrofuran (THF). To this solution, a mixture of 6 mL of a 3 M solution of methylmagnesium bromide (CH_3_MgBr) in diethyl ether was added (3:1 ratio), stirring overnight at room temperature. Silica gel column and thin layer chromatography (9:1 ethanol:methanol) were used to purify the main product. Once separated, the solvent was removed using a rotary evaporator and the product weighed. This intermediate, dissolved in dry THF, was slowly added to a suspension of lithium aluminium hydride (LiAlH_4_; 1:1 in mass), and the mixture was refluxed overnight. Once the reduction was complete, drops of saturated aqueous sodium hydroxide (NaOH) and isopropyl alcohol (IPA) were added to form a solid cake that was then washed with THF and filtered. The solvent (mainly THF) fraction was concentrated as before, and the amine residue was distilled (190°C at 10 Torr). The distilled product was neutralized using a saturated solution of hydrochloric acid in methanol to obtain a white solid (43% overall yield). ^1^H NMR (400 MHz, MeOH-d_4_) δ 7.25 (d, J = 8.4 Hz, 2H), 6.95 (d, J = 8.4 Hz, 2H), 3.80 (s, 3H), 3.162 (m, 2H), 3.02 (m, 1H), 1.34 (d, J = 6.4 Hz, 3H). ^13^C NMR (125 Hz, MeOH-d_4_, 300 K): δ 159.13, 133.54, 127.89, 114.15 (ArC), δ 54.37 (OCH_3_), δ 47.63 (CH_2_NH_2_), δ 37.36 (CH), δ 18.64 (CH_3_) ppm. HRMS for C_10_H_16_NO [M-Cl]^+^ m/z Calcd: 166.1226. Observed: 166.1223 (S2 Fig).

### Theoretical calculations

To obtain structural and energetic information regarding the MAO-B/p-CMP and MAO-B/p-MMP complexes, we applied the quantum chemical cluster approach for modeling enzyme reactions. This methodology considers a selected part of the enzyme (ca. 229 atoms for p-CMP, 233 atoms for p-MMP) provided that this small cluster behaves like the real system (S3 and S4 Figs), taking into account two the steric constraints imposed by the enzyme on the active site and long-range polarization effects. Steric constraints consider all the side chain Cαs of the cluster residues for MAO-B as locked into their crystallographic positions. Long range polarization effects take into account solvation effects using the CPCM conductor-like polarizable continuum model [33,34] with two dielectric constants, i.e. ε = 4 and ε = 80, performing single-point calculations on the optimized structures. The strength of this methodology makes the cluster selection independent of the dielectric constant [35]. Cluster selection was carried out using the crystal structure of human MAO-B expressed in *Pichia pastoris* complexed with p-nitrobenzylamine (NBA) (PDB code 2C70) [22]. NBA is bound to the binding site with its side chain pointing towards the FAD cofactor. Since no crystal structures are available for p-CMP and p-MMP, the pose adopted by NBA was used to locate their aromatic moieties. The active site models included the isoalloxazine ring, Tyr398 and Tyr435, Tyr188, Gln206, Lys296, Cys172, Ile199 and Tyr326 and six water molecules present in this crystal structure, further verified by the WaterDock [36] script in the Vina Docking program.

All calculations were carried out using the meta-hybrid GGA functional M06-2X [37] implemented in the Gaussian09 suite of programs [38]. Geometries were optimized using the 6-31G(d,p) basis set. In order to get more accurate energies, single-point calculations were carried out on the optimized geometries using the larger 6–311+G(2d,2p) basis set. Frequencies were computed analytically at the same level of theory as the geometry optimizations to confirm whether the obtained structures were minima or transition states and to obtain zero-point energy (ZPE) corrections. The final reported energies consist of the large basis set energies corrected for ZPE, solvation, and dispersion effects. In order to unequivocally assign reactants and products we scaled the transition state frequencies, which then were optimized and confirmed as local minima. Imaginary frequencies of the enzymatic transition states were -1153.3, and -1048.8 cm^-1^ for p-CMP and p-MMP, respectively.

The reaction rate constant (k) can be expressed as:

$$\text{k}=\frac{{\text{k}}_{\text{b}}\text{T}}{\text{h}}{\text{e}}^{\frac{-\Delta {\text{G}}^{\neq }}{\text{RT}}}$$ (1)

For enzymatic reactions, the calculated reaction barrier can be compared with the experimental kinetic results, provided *k*_*cat*_ is available. Thus, the experimental Gibbs free energy barrier was obtained from kinetic rate constants using Eq 2.

$$\Delta {\text{G}}_{\text{exp}}^{\neq }=-\text{RTln}\;\left(\frac{\text{kh}}{{\text{k}}_{\text{b}}\text{T}}\right)$$ (2)

where k = rate constant; k_b_ = Boltzmann constant; T = temperature; ΔG^≠^ = free energy of activation; h = Planck’s constant; R = gas constant. This equation is valid for simple transition state theory where the transmission coefficient is approximated to unity [39].

NCIPLOT software was used to understand the anchoring of the studied compounds at the active site. This program calculates the NCI indexes, which are based on the reduced density gradient defined as s(ρ) (Eq 3) at low densities and accounting for non-covalent interactions such as van der Waals, steric clashes and hydrogen bonds.

$$\text{s}=\frac{1}{2{\left(3\pi^{2}\right)}^{1/3}}\frac{\nabla \rho }{\rho^{4/3}}$$ (3)

In order to recognize the nature of the non-bonded interactions, the sign of the second eigenvalue (λ2) of the Laplacian of the density is related to: 1) hydrogen bonds if λ2 < 0, 2) steric repulsion if λ2 > 0 and 3) van der Waals interactions if λ2 ≈ 0. Therefore, ρ*sign(λ2) ranges from negative to positive values and the density itself is used to evaluate the strength of the interaction [40,41]. Analysis of NCI peaks is needed to differentiate between interaction types. The sign of the Laplacian of the density (∇^2^ρ) indicates whether the net gradient flux is entering (∇^2^ρ < 0) or leaving (∇^2^ρ > 0) an infinitesimal volume around a reference point. Hence, it highlights whether the density is concentrated or depleted at that point, relative to the surrounding environment [42].

## Results and Discussion

In order to study the influence of the para-substituent on the activity of β-methylphenylalkylamines, we prepared p-methoxy-β-methylphenethylamine (p-MMP) where the chlorine atom is replaced by a sterically similar methoxyl group on the aromatic ring. As mentioned before, Kinemuchi et al. and Kim et al. [30, 31] found that p-CMP was a highly selective MAO-B-inhibitor. However, we found that its p-methoxyl counterpart behaves like a selective substrate, with a low k_cat_ of 14 min^-1^ and a Michaelis constant of 87 μM compared to phenylethylamine (PEA) which is described as a good MAO-B substrate (K_cat_ 300 min^-1^ and K_m_ 0.016 mM) [21]. According to these experimental results, we hypothesize that the nature of the *para*-substituent determines the agonist or antagonist character displayed by these substrate analogues with MAO-B. Table 1 shows that p-chlorophenethylamine (p-CP), as a model of compounds lacking the β-methyl group, is a poor substrate with a K_cat_ of 3.41 min^-1^, and β-methylation (as in the case of p-CMP) turns this molecule into an inhibitor (K_i_ = 0.55 μM) as reported by Kinemuchi et al. [30]

**Table 1:** p-CMP and p-MMP kinetic constants for MAO-B and experimental and calculated free Gibbs energy barriers.

*Molecule*	K_i_ (μM)	k_cat_ (min^-1^)	K_m_ (μM)	ΔG^ǂ^_exp_^e^ (kcal/mol)	ΔG^ǂ^_theo_^e^ (kcal/mol)
*PEA*	N.D.	228 ± 0.9^a^	16 ± 1^a^	16.7^b^	17.1^b^
*p-CP*^c^	N.D.	3.41 ± 0.12	2.4 ± 0.2	19.15	N.C.
*p-CMP*	0.55^d^	N.D.	N.D.	N.D.	35.0
*p-MMP*	N.D.	14 ± 1	87 ± 8	18.3	19.5

^a^ Constants reported by Li et al. [21].

^b^ Constants reported by Zapata et al. [23].

^c^ Constants reported by Heuson et al. [43].

^d^ Constants reported by Kinemuchi et al. [30].

^e^ See Electronic Supplementary Information. N.D. = Not Determined. N.C. = Not Calculated.

**Fig 1:**
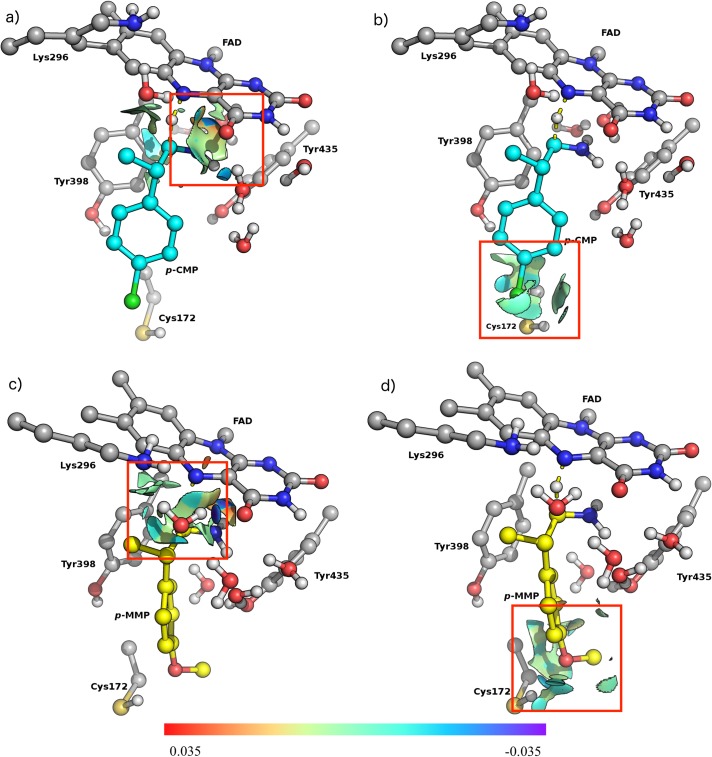
Non-covalent interaction (NCI) surface for binding site models in TS complexes with p-CMP and p-MMP after QM optimization. Red squares indicate hydrogen bonds (blue surfaces) and favorable van der Waals interactions (light green surfaces). a) the reactive region of p-CMP; b) p-substituent region of p-CMP, c) the reactive region of p-MMP and d) p-substituent region of p-MMP. p-CMP is depicted in cyan ball and sticks while p-MMP is depicted in yellow ball and sticks. NCI indexes isovalues range from 0.035 to -0.035 (au). p-MMP (p-methoxy-β-methylphenylethylamine); p-CMP (p-chloro-β-mehtylphenylethylamine), respectively.

We modelled the rate-determining step and calculated the reactant, transition state and product energies and free Gibbs energy barriers for the MAO-B/p-CMP and MAO-B/p-MMP complexes (S1 Table). All states were confirmed as ground or transition states according to their calculated frequencies. Our results show that the p-chloro derivative displays a higher activation energy compared to its p-methoxylated counterpart (35 kcal/mol vs 19.5 kcal/mol). The lower energy barrier displayed by p-MMP is in agreement with the obtained experimental value of 18.3 kcal/mol (Table 1). It is worth mentioning that energy barriers higher than 25 kcal/mol make enzyme catalysis very unlikely [44]. Also, NCI (non-covalent interaction) indexes were evaluated to provide information regarding weak ligand-enzyme interactions based on the reduced density gradient in low-density regions [40–42]. These interactions are important since they govern, not only the anchorage, but also the stabilization of the substrate orientations at the binding site. Inspection of the obtained TS geometries of both complexes (Fig 1) shows that the bound p-CMP ring is rotated by ca. 60° with respect to that of p-MMP.

This conformation is best explained by the non-covalent interactions established in the active site by p-CMP. Also, taking into account the anisotropic electronic distribution on the chlorine atom [45], the generation of a positive σ hole at the far end of the C-Cl bond allows a specific and orthogonal hydrogen bond to form. Specifically, a weak hydrogen bond can be seen between the chlorine atom of p-CMP and the amide moiety of Cys172 (S5 Fig). Fig 1 shows that the prevailing interactions for p-CMP are van der Waals in nature according to the NCI indexes (light green coloured).

On the other hand, the methoxyl oxygen atom of p-MMP also interacts with the thiol hydrogen of Cys172; however, its methyl group is located opposite to the position of the chloride atom of p-CMP (Fig 1B and 1D, and S6 Fig).

**Fig 2:**
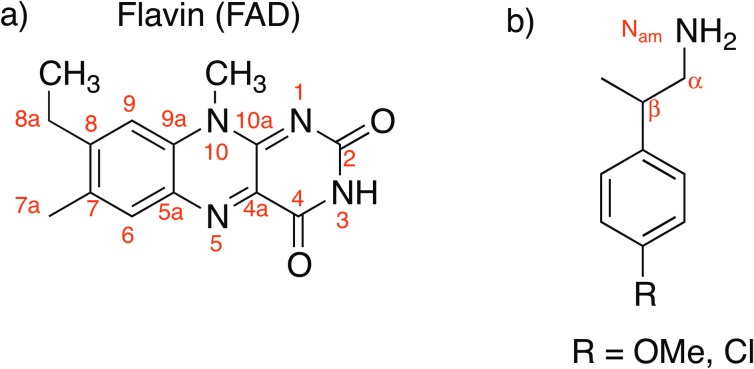
Structure and nomenclature. a) isoalloxazine ring of FAD. b) p-CMP or p-MMP.

The differences described above are directly related to the interactions established in the reactive region of the TS structures, which comprises N5 and C4a of FAD, and the amine nitrogen (Nam), Cα and Hα of the substrate (Fig 2). In Fig 1A and 1C it can be seen that p-MMP displays stronger interactions than p-CMP. Specifically, p-MMP exhibits a strong hydrogen bond between O4 of FAD and a conserved water molecule linking FAD to Lys296.

**Fig 3:**
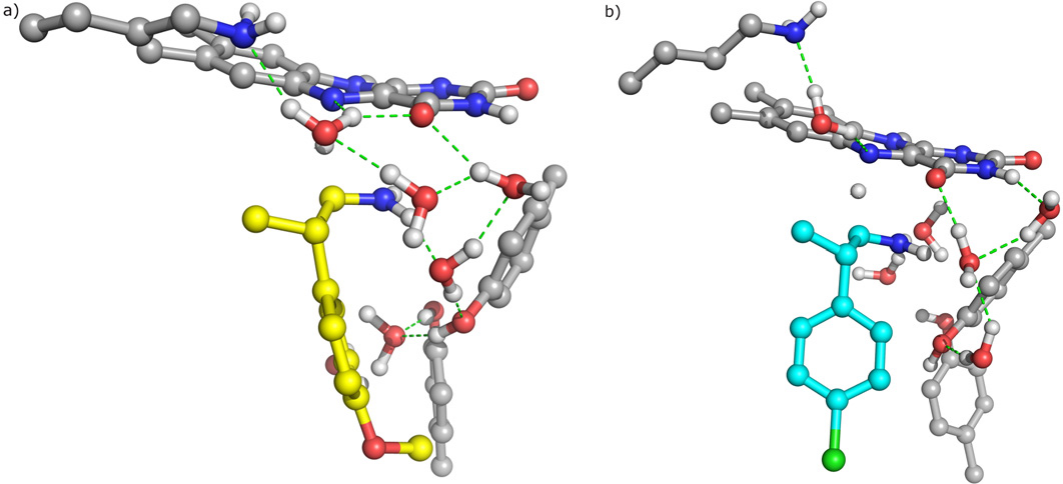
Hydrogen bond network. In a) p-CMP and b) p-MMP at the active site of MAO-B.

This water molecule has been reported to be important in catalysis, stabilising the TS by linking Lys296 to FAD [23]. Additionally, we note that both p-CMP and p-MMP display an interaction between Nam and C4a of the flavin, which has been suggested to occur in the polar nucleophilic mechanism (S7 Fig) [23]. Also, it should be emphasized that the TS of p-MMP is stabilized by a strong hydrogen bonding network of water molecules, which in the case of p-CMP is almost absent (Fig 3). This loss of hydrogen bonding interactions explains the calculated difference in the activation energies of both molecules (ca. 15 kcal/mol).

Our findings indicate that β-methyl substitution on phenethylamines allows substrates to adopt a suitable orientation to undergo catalysis; i.e. leaving the pro-R Hα pointing towards N5 of FAD. However, the other interactions established only by p-MMP allow the proper orientation of its aromatic ring parallel to the two tyrosine residues of the aromatic cage, thus mimicking other MAO-B substrates [23]. Therefore, conformational constraints imposed by β-methylation in combination with the restricted interaction displayed by the p-chlorine substituent and the breaking of the water network place p-CMP in the substrate pocket but with features that do not allow the oxidation to occur. These results are in agreement with previous conclusions of Edmondson et al. [46] who argue that the para-position of the bound substrate is in a hydrophobic domain of limited size. With these restrictions, bound p-MMP can adopt an orientation that favors its substrate character.

## Conclusions

In conclusion, although both p-CMP and p-MMP meet the structural requirements for MAO-B substrates, it is clear that the combined presence of a β-methyl group and para-substituents of similar volume but different electronic properties on the aromatic ring, determines the agonist or antagonist activity of these compounds. While experimental data for a larger set of compounds would most likely enrich this discussion, our experimental and theoretical results strongly suggest that the different activities displayed by these two very similar compounds depends on the conformation they can adopt upon binding in the MAO-B catalytic site.

## Supporting Information

S1 Fig HRMS-ESI spectrum. HRMS-ESI analyses were carried out by using a Thermo Scientific Exactive Plus Orbitrap spectrometer with a constant nebulizer temperature of 250° C. The experiment was carried out in positive ion mode at high resolution (resolving power: 140,000 (full width half-maximum peak width at *m*/*z* 300, R_fwhm_). The samples were infused directly into the ESI source using a syringe pump at flow rates of 5 μL min^-1^.
(PDF)

S2 Fig Plots of ^1^H and ^13^C NMR spectra. (PDF)

S3 Fig Cluster considered for the transition state structure for p-CMP. Atoms and bonds are depicted as balls and sticks. p-CMP in cyan. All other atoms are depicted as follows: carbon atoms in grey, oxygen atoms in red, nitrogen atoms in blue and hydrogen atoms in white.
(PDF)

S4 Fig Cluster considered for the transition state structure for p-MMP. Atoms and bonds are depicted as balls and sticks. p-MMP in yellow. All other atoms are depicted as follows: carbon atoms in grey, oxygen atoms in red, nitrogen atoms in blue and hydrogen atoms in white.
(PDF)

S5 Fig Full NCI indexes of the transition state of p-CMP at the active site. NCI indexes coloured ranging from 0.035 to -0.035 (au). For the sake of clarity some amino acid side-chains have been deleted from the figure.
(PDF)

S6 Fig Full NCI indexes of the transition state of p-MMP at the active site. NCI indexes isovalues coloured ranging from 0.035 to -0.035 (au). For the sake of clarity some amino acid side-chains have been deleted from the figure.
(PDF)

S7 Fig Proposed polar nucleophilic mechanism. This mechanism involves a proton transfer by means of a Nam-C4a adduct.
(PDF)

S1 Table Calculated contributions to free energy of activation for p-CMP and p-MMP complexed with MAO-B. (PDF)
