# Supplementary material for: Why p-OMe- and p-Cl-β-Methylphenethylamines Display Distinct Activities upon MAO-B Binding
Source: PLoS One. 2016 May 6;11(5):e0154989. doi: 10.1371/journal.pone.0154989 (PMC4859490; doi:10.1371/journal.pone.0154989)
Supplement: S1 Fig — HRMS-ESI analyses were carried out by using a Thermo Scientific Exactive Plus Orbitrap spectrometer with a constant nebulizer temperature of 250° C. The experiment was carried out in positive ion mode at high resolution (resolving power: 140,000 (full width half-maximum peak width at m/z 300, Rfwhm). The samples were infused directly into the ESI source using a syringe pump at flow rates of 5 μL min-1. (PDF) [file pone.0154989.s001.pdf]

S1 Fig. **HRMS-ESI spectrum**. HRMS-ESI analyses were carried out by using a Thermo Scientific Exactive Plus Orbitrap spectrometer with a constant nebulizer temperature of 250° C. The experiment was carried out in positive ion mode at high resolution (resolving power: 140,000 (full width half-maximum peak width at *m*/*z* 300, R_{fwhm}). The samples were infused directly into the ESI source using a syringe pump at flow rates of 5 μL min^{-1}.

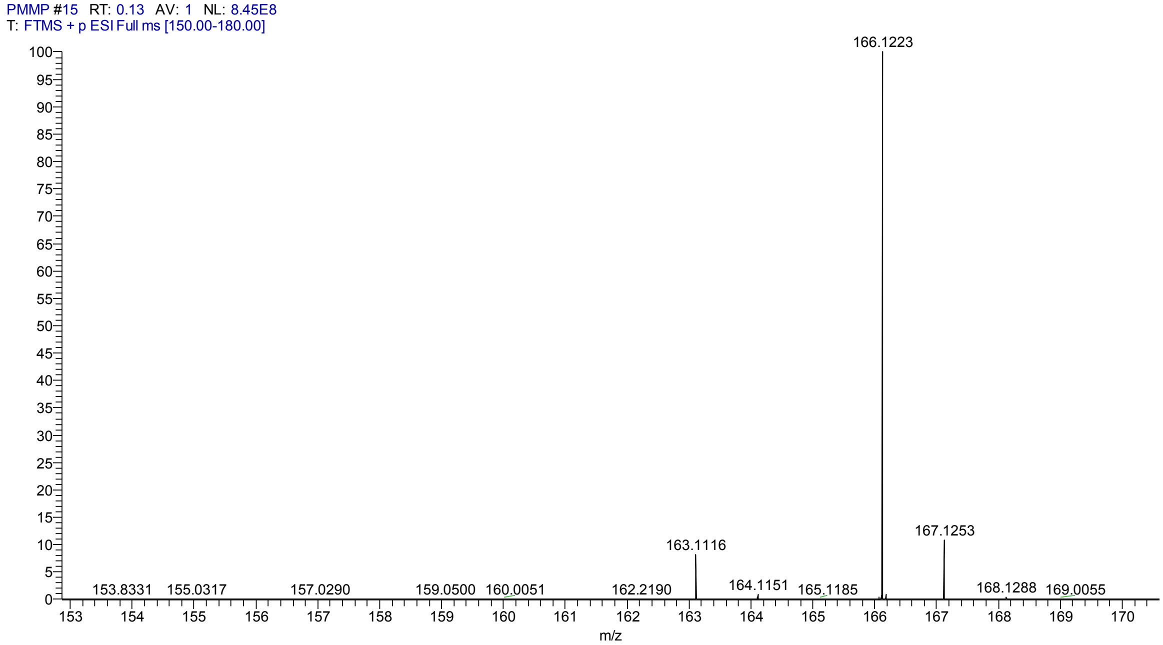

2-(4-methoxyphenyl)propan-1-aminium chloride;

HRMS for $C_{10}H_{16}NO$ $[M\text{-}Cl]^+$ m/z Calcd: 166.1226. Observed: 166.1223
