# Supplementary material for: Why p-OMe- and p-Cl-β-Methylphenethylamines Display Distinct Activities upon MAO-B Binding
Source: PLoS One. 2016 May 6;11(5):e0154989. doi: 10.1371/journal.pone.0154989 (PMC4859490; doi:10.1371/journal.pone.0154989)
Supplement: S2 Fig — (PDF) [file pone.0154989.s002.pdf]

**S2 Fig. Plots of $^{1}H$ and $^{13}C$ NMR spectra.**

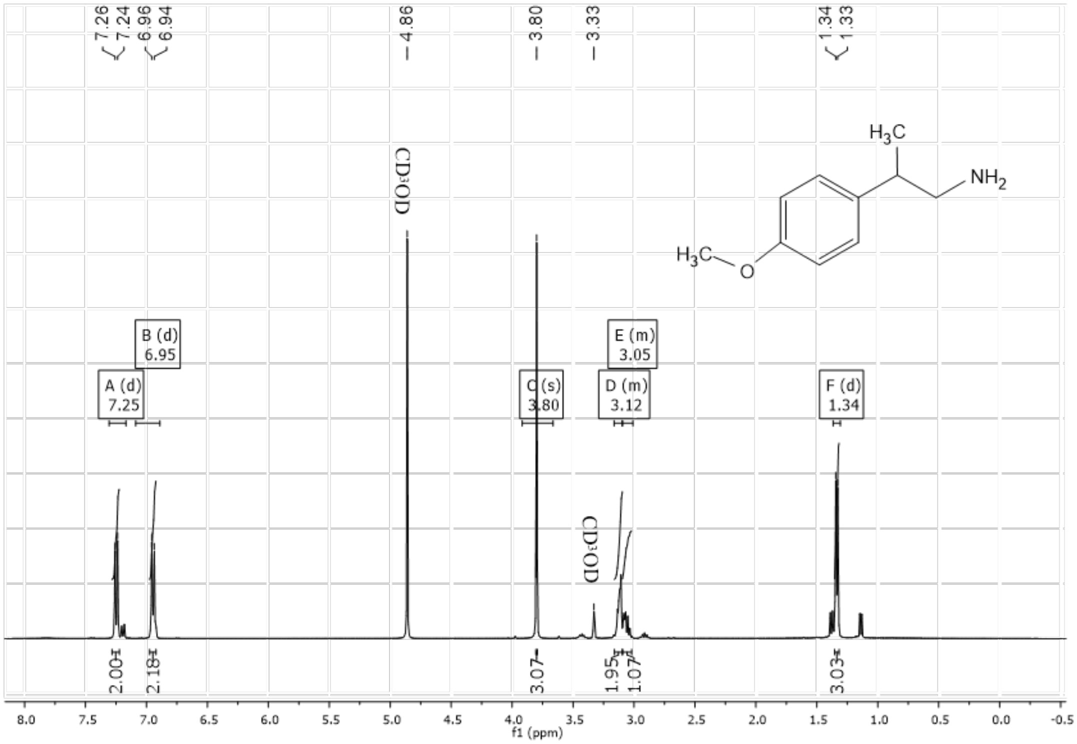


$^{1}H$ NMR (400 MHz, MeOH-$d_4$)

**$^{1}$H NMR** (400 MHz, MeOH-$d_4$ ) δ 7.25 (d, $J$ = 8.4 Hz, 2H), 6.95 (d, $J$ = 8.4 Hz, 2H), 3.80 (s, 3H), 3.162 (m, 2H), 3.02 (m, 1H), 1.34 (d, $J$ = 6.4 Hz, 3H).

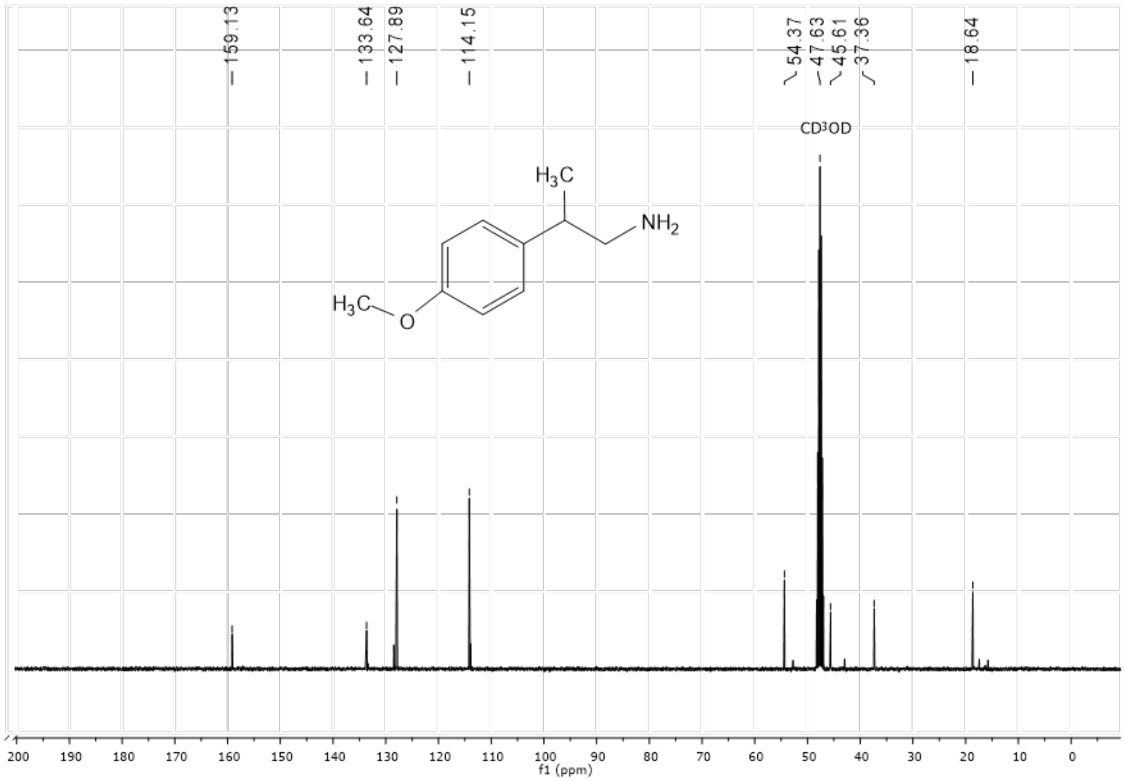


$^{13}$C NMR (125 MHz, MeOH-$d_4$)

**$^{13}$C NMR** (125 Hz, MeOH-$d_4$, 300 K): δ 159.13, 133.54, 127.89, 114.15 (ArC), δ 54.37 ($OCH_3$), δ 47.63 ($CH_2NH_2$), δ 37.36 (CH), δ 18.64 ($CH_3$) ppm.
