# Supplementary material for: Why p-OMe- and p-Cl-β-Methylphenethylamines Display Distinct Activities upon MAO-B Binding
Source: PLoS One. 2016 May 6;11(5):e0154989. doi: 10.1371/journal.pone.0154989 (PMC4859490; doi:10.1371/journal.pone.0154989)
Supplement: S4 Fig — Atoms and bonds are depicted as balls and sticks. p-MMP in yellow. All other atoms are depicted as follows: carbon atoms in grey, oxygen atoms in red, nitrogen atoms in blue and hydrogen atoms in white. (PDF) [file pone.0154989.s004.pdf]

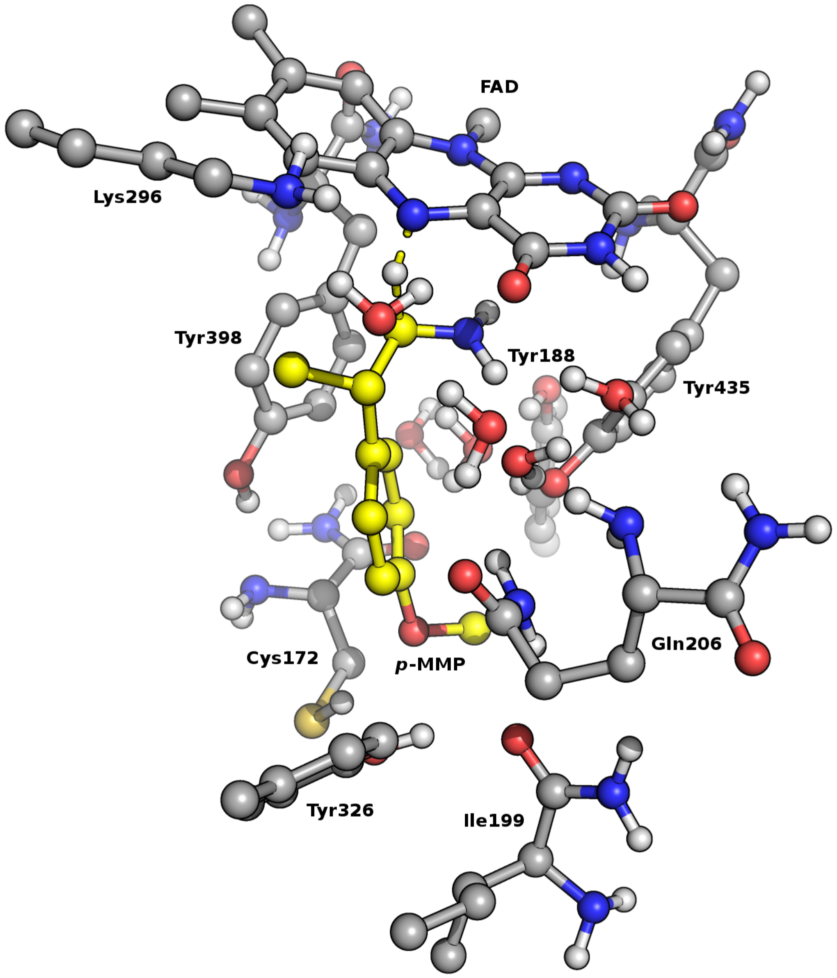

S4 Fig. Cluster considered for the transition state structure for p-MMP. Atoms and bonds are depicted as balls and sticks. p-MMP in yellow. All other atoms are depicted as follows: carbon atoms in grey, oxygen atoms in red, nitrogen atoms in blue and hydrogen atoms in white.
