# Supplementary material for: Why p-OMe- and p-Cl-β-Methylphenethylamines Display Distinct Activities upon MAO-B Binding
Source: PLoS One. 2016 May 6;11(5):e0154989. doi: 10.1371/journal.pone.0154989 (PMC4859490; doi:10.1371/journal.pone.0154989)
Supplement: S6 Fig — NCI indexes isovalues coloured ranging from 0.035 to -0.035 (au). For the sake of clarity some amino acid side-chains have been deleted from the figure. (PDF) [file pone.0154989.s006.pdf]

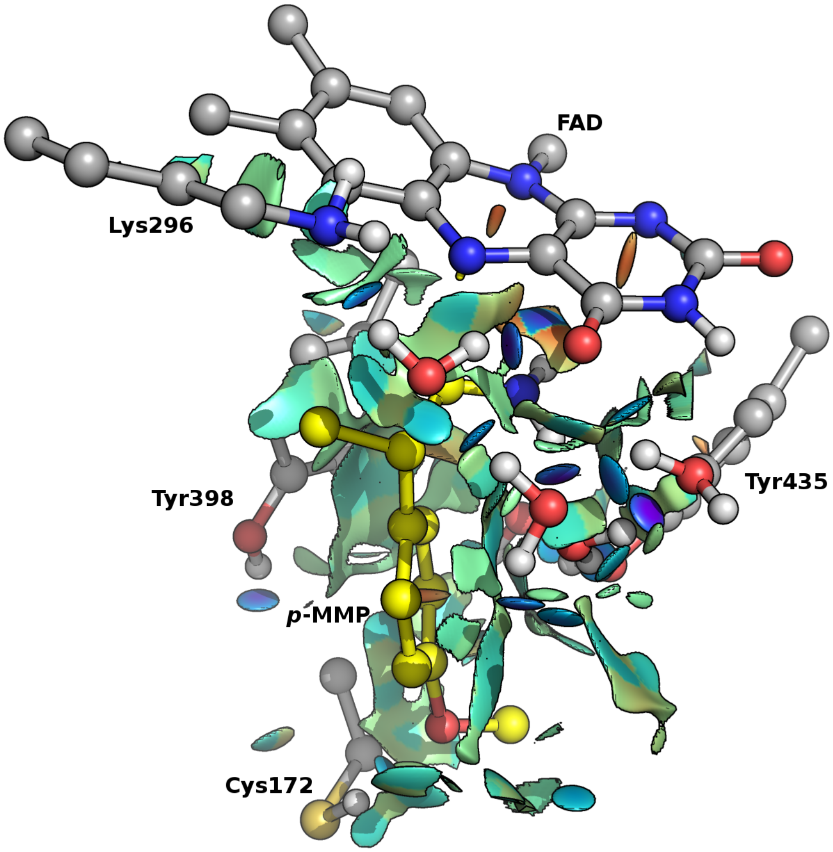

S6 Fig. Full NCI indexes of the transition state of p-MMP at the active site. NCI indexes isovalues colored ranging from 0.035 to -0.035 (au). For the sake of clarity some amino acid side-chains have been deleted from the figure.
