# Supplementary figures and images for: Why p-OMe- and p-Cl-β-Methylphenethylamines Display Distinct Activities upon MAO-B Binding
Source: PLoS One. 2016 May 6;11(5):e0154989. doi: 10.1371/journal.pone.0154989 (PMC4859490; doi:10.1371/journal.pone.0154989)

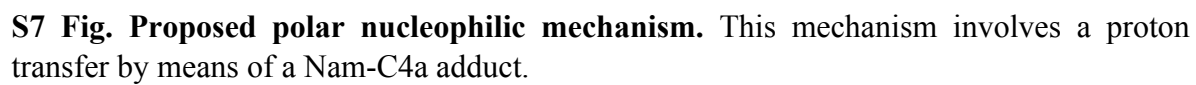

Supplement: S7 Fig — This mechanism involves a proton transfer by means of a Nam-C4a adduct. (PDF) [file pone.0154989.s007.pdf]
