# Supplementary material for: Why p-OMe- and p-Cl-β-Methylphenethylamines Display Distinct Activities upon MAO-B Binding
Source: PLoS One. 2016 May 6;11(5):e0154989. doi: 10.1371/journal.pone.0154989 (PMC4859490; doi:10.1371/journal.pone.0154989)
Supplement: S1 Table — (PDF) [file pone.0154989.s008.pdf]

Table S1. Calculated contributions to free energy of activation for *p*-CMP and *p*-MMP complexed with MAO B.

Substrate	Complex State	[a]BBS	[b]Solvation Effects		[c]Thermal Correction and ZPE	[d]Free Gibbs Energy		[e]$\Delta G^{\neq}_{\varepsilon=4}$	[e]$\Delta G^{\neq}_{\varepsilon=80}$
			$\varepsilon = 4$	$\varepsilon = 80$		$\varepsilon = 4$	$\varepsilon = 80$		
***p*-CMP**	RC	-5959.0633566	0.2196339	0.2918863	1.767073	-5957.0766497	-5957.0043973	35.0	34.6
	TS	-5959.0062118	0.2175651	0.2891722	1.767758	-5957.0208887	-5956.9492816		
***p*-MMP**	RC	-5613.9583915	0.2295289	0.3019354	1.819695	-5611.9091676	-5611.8367611	19.5	20.2
	TS	-5613.9128681	0.2216267	0.2951898	1.81311	-5611.8781314	-5611.8045683		

[a]Single point energy in hartrees at M06-2X/6-311+(2d,2p) level of theory (Big Basis Set).
[b]Solvent contribution to the free Gibbs energy in hartrees calculated using ε=4 and ε=80.
[c]Thermal correction to the free Gibbs energy and Zero Point Correction in hartrees.
[d]Sum of BBS+Solvation+Thermal corrections terms in hartrees.
[e]Difference between TS and RC free Gibbs energies in kcal/mol.
*Correspond to G_{Adduct}-G_{RC} in kcal/mol.
